# Frequency of scapular dyskinesis and its relationship with disease parameters in patients with ankylosing spondylitis: a cross-sectional study

**DOI:** 10.1590/1516-3180.2024.0136.R1.13082024

**Published:** 2025-04-28

**Authors:** Sertaç Ketenci, Bora Uzuner, Dilek Durmuş, Deniz Şahinkaya, Muharrem Yüksel, Ahmet Kıvanç Cengiz

**Affiliations:** I Department of Physical Medicine and Rehabilitation, Department of Rheumatology, Medical Faculty, Ondokuz Mayıs University, Samsun, Turkey.; II Department of Physical Medicine and Rehabilitation and Department of Algology, Medical Faculty, Ondokuz Mayıs University, Samsun, Turkey.; III Department of Physical Medicine and Rehabilitation, Medical Faculty, Ondokuz Mayıs University, Samsun, Turkey.; IV Department of Physical Medicine and Rehabilitation, Medical Faculty, Ondokuz Mayıs University, Samsun, Turkey.; V Department of Physical Medicine and Rehabilitation, Medical Faculty, Ondokuz Mayıs University, Samsun, Turkey.; VI Department of Physical Medicine and Rehabilitation and Department of Rheumatology, Medical Faculty, Ondokuz Mayıs University, Samsun, Turkey.

**Keywords:** Scapula, Dyskinesis, Spondylitis, ankylosing, Arthritis, Juvenile, Spondyloarthritis, Scapular dyskinesis, Posture and scapula, Seronegative arthritis, Shoulder pain

## Abstract

**BACKGROUND:**

Scapular dyskinesis (SD) is a condition associated with impaired scapular movement caused by cervical, shoulder, and postural abnormalities.

**OBJECTIVE:**

The aim of this study was to determine the frequency of SD in patients with ankylosing spondylitis (AS).

**DESIGN AND SETTING:**

A cross-sectional study was conducted at Ondokuz Mayıs University, Samsun, Turkey.

**METHODS:**

One hundred patients with AS but without shoulder involvement (74 males and 26 females) and 50 healthy controls (35 males and 15 females) were included in the study. The patients were divided into two groups: patients with and without SD. SD was assessed using the Scapular Dyskinesis Test and Lateral Scapular Slide Test. Disease activity, spinal mobility, and chest expansion were also measured. The severity of enthesitis was evaluated using the Spondyloarthritis Research Consortium of Canada index.

**RESULTS:**

There were significant differences between the two groups of patients with AS, those with SD, and those without SD in terms of age, chest expansion, and the Bath Ankylosing Spondylitis Metrology Index (BASMI) scores (P < 0.05). The groups differed significantly in terms of hip, thoracic, and lumbar involvement (P < 0.05). The BASMI score was a significant variable affecting SD (P < 0.05). No cases of SD were observed in the control group.

**CONCLUSION:**

While there were no significant differences in disease activity and enthesitis scores between patients with and without SD, differences were detected in mobility parameters. Since shoulder examinations of the patients were normal, it can be inferred that SD occurred because of the involvement of the scapulothoracic joints and thoracic spine.

## INTRODUCTION

Ankylosing Spondylitis (AS) is a chronic inflammatory disease primarily affecting the spine and peripheral joints. The course of AS may be progressive, starting at the sacroiliac joints and leading to ankylosis throughout the spine, including the cervical region. The development of ankylosis can restrict joint mobility, causing significant disability and deterioration in the quality of life. In cases of extra-spinal involvement, one of the target joints is the shoulder joint, where inflammation can cause severe pain and restrict shoulder movement.^1^ Previous studies have reported that the frequency of shoulder involvement in patients with AS varies between 3.5% and 33%.^2^

Coordinated movement of the glenohumeral joint and scapula is crucial for proper shoulder function. The scapula plays an important role in the stability of the shoulder joint.^3^ Changes in scapular position and problems in scapulothoracic joint movement can lead to shoulder pathologies.^4^ Therefore, the examination of scapular functions is crucial in the evaluation of patients with shoulder complaints.

Scapular dyskinesis (SD) is defined as the deviation of the scapula from its normal position at rest and/or during movement.^5^ Previous studies have demonstrated a high prevalence of SD in individuals with shoulder pathologies such as rotator cuff problems and impingement syndrome, as well as in elite athletes who frequently use their shoulders.^6^ Any pathology affecting the glenohumeral or scapulothoracic joint is likely to be associated with SD. In patients with AS, kyphosis, shoulder involvement with arthritis, and enthesitis can impair scapulothoracic movements. To our knowledge, the presence of SD in patients with AS has not been previously evaluated.

## OBJECTIVE

This study aimed to investigate the frequency of SD in patients with AS, particularly in those without shoulder involvement. In addition, possible associations between SD and disease activity, spinal mobility, enthesitis, and chest expansion were assessed.

## METHODS

A total of 112 consecutive patients with axial spondyloarthritis (ax-SpA) who were treated at the Rheumatology Clinic of Ondokuz Mayıs University Faculty of Medicine fulfilled the Assessment of SpondyloArthritis International Society (ASAS) classification criteria for ax-SpA, and had current sacroiliac and spinal radiographs revealing radiological sacroiliitis consistent with AS were enrolled in the study. Patients with scoliosis or shoulder and neck pathologies that could cause SD were excluded from the study. Patients with a history of psychiatric or rheumatological diseases other than AS were also excluded. All participants underwent shoulder ultrasonography to identify shoulder pathologies, such as arthritis, enthesitis, shoulder impingement syndrome, or rotator cuff tears. Although these findings did not result in obvious clinical symptoms, the patients with sonographic findings were excluded. After applying these criteria, 12 patients were excluded, and the study was conducted on the remaining 100 patients.

The control group included 50 healthy individuals aged > 18 years, consisting of relatives of patients from outpatient clinics, university employees, and volunteers from the general population.

This study was performed in accordance with the Declaration of Helsinki, and the protocol was approved on December 23, 2023, by the Medical Research Ethics Committee at Ondokuz Mayıs University (No:289/2023). The patients and controls were informed of the study, and written consent was obtained. Detailed physical examinations of the patients and control subjects were performed by the same PMR specialist with more than 20 years of experience. (DD).

A priori power analysis was performed, and the minimum sample size was determined to be 26 for each group.^7^ Demographic data (including age, sex, marital status, and disease duration) were also recorded.

### Clinical assessments

#### Socio-demographic characteristics

Age, sex, disease duration, age at diagnosis, drug usage (disease-modifying anti-rheumatic drugs and biological agents), history of peripheral arthritis, and extra-articular manifestations such as uveitis, inflammatory bowel disease, and psoriasis were noted for each patient.

All patients underwent standard physical examinations and anthropometric measurements. Only patients with radiographic sacroiliitis were included. Involvement of the lumbar, thoracic, and cervical spines was assessed by examining current radiographs for the presence of syndesmophytes, erosions, or ankylosis. The decision regarding the radiographic findings was obtained via consensus of two rheumatologists (SK and KC).

### Disease Activity

Clinical disease activity was evaluated using the Bath Ankylosing Spondylitis Disease Activity Index (BASDAI), Ankylosing Spondylitis Disease Activity Score-C-Reactive Protein (ASDAS-CRP), and ASDAS-Erythrocyte Sedimentation Rate (ASDAS-ESR):^8, 9, 10^

### Mobility

Patient mobility was evaluated using the Bath Ankylosing Spondylitis Metrology Index (BASMI), which includes measurements of wall-to-tragus distance, lumbar flexion, cervical rotation, lumbar lateral flexion, and intermalleolar distance. Chest expansion was measured with a tape measure placed circumferentially around the chest wall in the fourth intercostal space.^11, 12, 13^

### Enthesitis score

The severity of enthesitis was assessed via the Spondyloarthritis Research Consortium of Canada (SPARCC) enthesitis index evaluating tenderness in a total of 16 enthesitis sites: the greater trochanter (right/left [R/L]), quadriceps tendon insertion to the patella (R/L), patellar ligament insertion into the patella and tibial tuberosity (R/L), Achilles tendon insertion (R/L), plantar fascia insertion (R/L), medial and lateral epicondyles (R/L), and the supraspinatus insertion (R/L). Tenderness at each site was quantified on a dichotomous basis as follows: 0 = non-tender and 1 = tender. All parameters were evaluated by the same physician.^14^

### Evaluation of scapular dyskinesia

In the observational SD assessment using the Scapular Dyskinesis test (SDT), the patients were instructed to perform bilateral shoulder elevation movements consecutively 3–5 times in the sagittal plane. The medial border of the scapula was observed throughout the movement. The presence of a distinct prominence of the inferomedial, entire medial, or superior scapular border during movement was noted as SD. If none of these were observed, the presence of SD was ruled out. As shown in the literature, the test demonstrates high inter-rater agreement and sensitivity in the evaluation of SD.^15^

**Figure 1:**
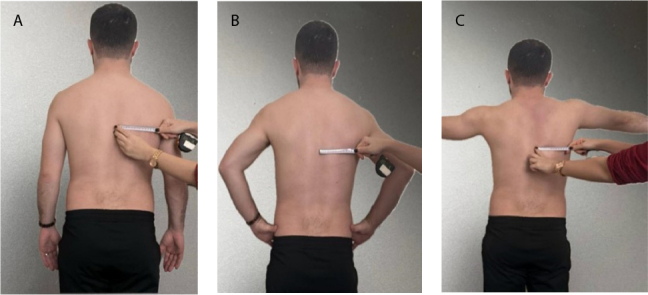
Measurement of the scapular position in the Lateral Scapular Slide Test.

The Lateral Scapular Slide Test (LSST) was performed with the patient in an upright position. Three different arm positions were examined: a) arms at the sides of the body (Figure 1a), b) hands on top of the hips (Figure 1b), and c) arms at 90º abduction (Figure 1c). The distance between the spinous processes of the vertebrae at the same level as the lower scapular angle was measured bilaterally using calipers. If the difference between measurements in the same position was greater than 1.5 cm, it was recorded as SD.^16^ Patients with positive results in both observational SDT and LSST assessments were recorded as having SD.

a) With arms resting at the sides, b) with hands placed on the hips, and c) with arms in 90º abduction.

Based on the LSST results, the side farther from the center was the asymmetric side, and the opposite side was the symmetric side.

### Statistical analyses

Statistical analyses were performed using SPSS software (version 21.0; IBM, Armonk, New York, United States). An a priori power analysis was performed, and the minimum sample size was determined to be 26 for each group. Descriptive statistics are presented as mean ± standard deviation, minimum-maximum, frequency distribution, and percentage. The Kolmogorov-Smirnov test was used to analyze the normal distribution assumption of the quantitative outcomes. Student’s t-test, Mann–Whitney U-test, and chi-square test were used to compare the clinical scores and demographic characteristics of the two groups. Intergroup comparison of the lateral scapular shift test was performed using Kruskal-Wallis analysis and Mann-Whitney U tests. Binary logistic regression analysis was performed to determine whether age, disease duration, sex, BASMI score, chest expansion, enthesitis score, ASDAS-ESR, ASDAS-CRP level, thoracic involvement, lumbar involvement, and sacroiliitis were associated with SD. P values less than 0.05 were considered statistically significant.

## RESULTS

Initially, 112 consecutive patients with AS but without known shoulder involvement were included in this study. After a detailed examination, 12 patients were excluded from the study (six had impingement syndrome, three had cervical disc herniation, one had a history of labral tear, one had a previous humerus fracture, and one was a basketball player prone to shoulder trauma). One hundred patients with AS and 50 healthy controls were included in this study. The age of those in the AS and control groups was 47.00 ± 11.84 (range: 21-63) and 44.00 ± 7.24 (range: 28-58), respectively, with no significant difference between the two groups (P = 0.062). The patient group comprised 74 males and 26 females, whereas the control group consisted of 35 males and 15 females, with no significant sex differences between the two groups (P = 0.604).

All participants underwent SDT and LSST. No participant in the control group had SD. Based on the test results, 26 patients with SD were categorized into Group 1, 74 patients without SD into Group 2, and healthy individuals into Group 3. The side farther from the center was considered asymmetric, and the opposite side was regarded as the symmetric side in the LSST assessment. In the comparison of the LSST results among the three groups, a significant difference was found on the asymmetric side in the three arm positions (P < 0.05). For the pairwise comparison of the groups, the LSST results for Group 1 and Group 2 are summarized in Table 1. A significant difference was observed in the asymmetric side between the two groups (P < 0.05). Although a significant difference was found between Groups 1 and 3 on the asymmetric side (P < 0.05), no significant difference was detected between Groups 2 and 3 for all measurements (P > 0.05).

**Table 1:** Comparison of demographic and clinical data of patients according to scapular dyskinesis

	Group 1 (n = 26) Mean ± SD (range)	Group 2 (n = 74) Mean ± SD (range)	P
Age (years)	51.30 ± 7.87 (37-63)	45.48 ± 12.65 (21-70)	0.028*
Disease duration (years)	15.03 ± 9.34 (2-34)	11.38 ± 8.91 (1-45)	0.081
Age at diagnosis (years)	33.30 ± 16.35 (12-59)	32.73 ± 12.50 (7-55)	0.436
Enthesitis score	1.07 ± 0.98 (0-6)	0.50 ± 0.33 (0-2)	0.763
BASMI	7.12 ± 5.12 (0.9-32)	2.48 ± 1.21 (0.5-6)	0.001*
Chest expansion (cm)	3.11 ± 1.13 (1-4.5)	4.27 ± 1.42 (2-6)	0.001*
ASDAS-ESR	1.96 ± 0.93 (1-4)	2.57 ± 1.18 (0-5)	0.259
ASDAS-CRP	1.71 ± 1.11 (0-3.6)	2.28 ± 1.17 (0-4.4)	0.336
BASDAI (n)			
≥ 4	3 (11.5)	18 (24.3)	0.169
4 >	23 (88.5)	56 (75.5)	
Gender			
Female	1 (3.9)	25 (33.8)	0.003*
Male	25 (96.1)	49 (66.2)	

*P < 0.05 significant; BASMI = Bath Ankylosing Spondylitis Metrology Index; ASDAS-CRP = Ankylosing Spondylitis Disease Activity Score-C-Reactive Protein; ASDAS-ESH = Ankylosing Spondylitis Disease Activity Score – Erythrocyte Sedimentation Rate; BASDAI = Bath Ankylosing Spondylitis Disease Activity Index; Group 1 = patients with scapular dyskinesis; Group 2 = patients without scapular dyskinesis.

Upon examining other patient data, it was found that the age of the patients in Group 1 was significantly higher (P < 0.05). However, there was no significant difference in disease duration between Groups 1 and 2 (P > 0.05) (Table 2). The BASMI scores were higher in Group 1, and the frequency of lumbar and thoracic involvement was significantly higher. Additionally, chest expansion was more restricted, and the percentage of hip involvement was higher in Group 1 (P < 0.05) (Table 2 and Table 3).

**Table 2:** Results of the Lateral Scapular Slide Test

	Group 1 (n = 26 Mean ± SD (min-max)	Group 2 (n = 74 Mean ± SD (min-max)	P
Position 1 Other side	9.20 ± 0.98 (6.7-11)	8.23 ± 2.55 (3.4-11.8)	0.571
Position 1 Asymmetrical side	10.42 ± 0.97 (8.2-12)	8.46 ± 2.53 (3.6-11.8)	0.002*
Position 2 Other side	9.34 ± 0.78 (7.4-10.5)	8.78 ± 2.43 (4-12.8)	0.634
Position 2 Asymmetrical side	10.76 ± 1.20 (7.8-12)	9.02 ± 2.37 (4.1-12.8)	0.001*
Position 3 Other side	10.03 ± 1.44 (6.9-13)	9.39 ± 2.38 (4.8-13.6)	0.939
Position 3 Asymmetrical side	10.92 ± 1.15 (8.5-13)	9.59 ± 2.35 (4.9-13.8)	0.033*

Position 1 = arms next to the body; Position 2 = hands on the hips; Position 3 = arms in 90º abduction; Group 1 = Patients with scapular dyskinesis; Group 2 = Patients without scapular dyskinesis.

**Table 3:** Comparisons of patients’ clinical parameters

		Group 1 (n = 26) Mean ± SD (min-max)	Group 2 (n = 74) Mean ± SD (min-max)	P
Lumbar involvement	Yes	8 (30.7)	9 (12.2)	0.030*
	No	18 (69.3)	65 (87.8)	
Thoracal involvement	Yes	7 (26.9)	7 (9.5)	0.036*
	No	19 (73.1)	67 (90.5)	
Cervical involvement	Yes	4 (15.4)	4 (5.4)	0.107
	No	22 (84.6)	70 (94.6)	
Hip involvement	Yes	10 (38.5)	7 (9.5)	0.001*
	No	16 (61.5)	67 (90.5)	
Heel enthesitis	Yes	4 (15.4)	11 (14.9)	0.949
	No	22 (84.6)	63 (85.1)	
Sacroiliitis (n%) Early stage (I-II)		12 (46.2)	51 (68.9)	0.039*
Late stage (III-IV)		14 (53.8)	23 (31.1)	
Uveitis (n%)	Yes	3 (11.5)	11 (14.9)	0.674
	No	23 (88.5)	63 (85.1)	
DMARDs (n%)	Yes	2 (15.4)	5 (13.2)	0.840
	No	11 (84.6)	33 (86.8)	
Biological agents ( n%)	Yes	13 (100)	36 (94.7)	0.399
	No	0	2 (5.3)	

DMARDs = Disease-modifying anti-rheumatic drugs; Group 1 = Patients with scapular dyskinesis; Group 2 = Patients without scapular dyskinesis.

In the assessments based on radiographs, when patients were categorized into mild stages (≤ 2) or advanced stages (3 or 4) of sacroiliitis, it was observed that in Group 1, sacroiliac involvement was in a more advanced stage. There were no significant differences between the two groups in terms of disease activity scores or medications used at the time of evaluation (P > 0.05). Detailed findings are provided in Table 2 and Table 3.

The relationships between clinical parameters and SD, as determined by binary logistic regression analysis, are presented in Table 3. The BASMI score was identified as a significant variable affecting SD (P < 0.05) (Table 4).

**Table 4:** Binary logistic regression analysis of factors associated with scapular dyskinesis

	B	Std. error	Wald	P	Exp (B)	95%CI for EXP(B)	
						Lower	Upper
Age	0.056	0.033	2.947	0.086	0.946	0.887	1.008
Sex	-2.469	1.319	3.506	0.061	0.085	0.006	1.122
Disease duration	0.046	0.037	1.544	0.214	1.047	0.974	1.125
Lumbar involvement	0.455	0.982	0.214	0.643	1.576	0.230	10.805
Thoracal involvement	1.055	1.101	0.918	0.338	2.871	0.332	24.833
Sacroiliitis	0.718	0.630	1.297	0.255	2.050	0.596	7.052
Enthesitis score	0.078	0.373	0.044	0.833	0.925	0.446	1.919
BASMI score	0.821	0.272	9.083	0.003*	0.440	0.258	0.750
Chest expansion	-0.124	0.079	2.439	0.118	0.883	0.756	1.032
ASDAS-ESR	0.343	0.646	0.283	0.595	1.410	0.397	5.002
ASDAS-CRP	0.009	0.561	0.000	0.987	0.991	0.330	2.974

*P < 0.05 significant; BASMI = Bath Ankylosing Spondylitis Metrology Index; ASDAS-CRP = Ankylosing Spondylitis Disease Activity Score-C-Reactive Protein; ASDAS-ESH = Ankylosing Spondylitis Disease Activity Score-Erythrocyte Sedimentation Rate; CI = confidence interval; Exp(B) = exponential of the B coefficient.

## DISCUSSION

Our study revealed a significant prevalence of SD in patients with AS through the implementation of SDT and LSST. Another noteworthy aspect of this study was the absence of shoulder involvement related to AS in all the participants. Our data support the notion that a substantial portion of SD can be observed in patients with AS but without arthritis and is solely associated with axial involvement.

Various pathologies have been implicated in the development of SD. Primarily, neck problems, shoulder-related factors, posture-related factors, and peripheral nerve damage can give rise to SD, as highlighted by Panagiotopoulos et al.^17^ Among those, a closer examination should particularly focus on shoulder joint pathologies, peripheral nerve damage innervating shoulder muscles, and pathologies related to cervical roots innervating shoulder muscles.^18^

Studies have demonstrated an increased frequency of SD in individuals with neck pain.^19^ Additionally, long thoracic nerve injury has been identified as another significant cause of SD and medial scapular winging, particularly resulting from overhead weightlifting.^20^ In our study, patients with neck pathologies were excluded. Physical examination of all patients was conducted for signs suggestive of root compression. While obtaining magnetic resonance imaging (MRI) to completely rule out cervical root compression could be suggested for thorough exclusion, it is important to note that the underlying mechanisms leading to SD in cervical pathologies often involve muscle spasms or weakness due to root compression. Patients were thoroughly questioned and evaluated through physical examinations in this regard. Therefore, we did not consider an underlying cervical mechanism or peripheral nerve damage as the cause of SD in our patient group.

Other pathologies that contribute to SD are shoulder-related. Acromioclavicular joint disorders, impingement syndrome, rotator cuff and glenoid labrum pathologies, and clavicle fractures are the main shoulder pathologies associated with SD.^3,17^ In a study by Christiansen et al. involving 40 patients with impingement syndrome, they demonstrated the presence of SD in nearly half of the patients before treatment.^21^ In their study, Keshavarz et al. noted that patients with shoulder impingement syndrome exhibited increased scapular protraction at rest, increased posterior tilt during abduction, and increased internal rotation during elevation.^22^ Similarly, in a study involving patients with a history of shoulder fractures, Suphakitchanusan et al. found the frequency of SD to be 50%.^23^ Therefore, in our study, shoulder pathologies that could cause SD were excluded through medical history, examination, and ultrasonographic evaluation.

It is worth noting that SD can be observed in individuals with excessive use of the upper extremities, even in the absence of any shoulder pathology, particularly in athletes with frequent overhead movements.^24^ Even in the absence of shoulder pathology, fatigue-related weakness in the rotator cuff, scapula stabilizers, and latissimus dorsi muscles due to chronic use appears to be the main underlying mechanism. In a study by Zago et al., an isokinetic fatigue protocol was applied to 24 healthy professional overhead athletes, and measurements were performed before and after the protocol. When evaluating the pre-post fatigue range of motion, they observed a significant decrease in shoulder elevation and a significant increase in scapular tilt. Computer-assisted measurements revealed a significant delay in initiating humeroscapular movement after fatigue, indicating that fatigue leads to significant changes in scapulohumeral rhythm.^25^ None of the patients were engaged in professional sports, and when occupations involving overhead limb usage were queried, none of the patients reported such activities. Furthermore, as all potential shoulder pathologies were ruled out through physical examination and ultrasonographic assessment, we did not consider SD to be primarily associated with shoulder joint dysfunction.

Postural abnormalities are implicated in the emergence of SD in individuals, excluding cervical or shoulder-related pathologies.^17^ Prolonged postures that place excessive strain on the shoulder girdle may exacerbate these biomechanical issues, further promoting the development of SD. The scapulothoracic joint is involved in shoulder movement. SD can arise in any condition affecting the scapula and the thoracic part of the scapulothoracic surface. For a normal movement pattern, it is necessary for the thorax to have a normal ellipsoid structure and the kyphosis angle to be within normal limits.^26^ Especially in situations where thoracic kyphosis increases, which can be a contributing factor to the occurrence of SD. In a study conducted by Telli et al. on patients with myofascial pain syndrome, SD was significantly more prevalent in individuals with increased kyphosis.^27^ In another study conducted by Otoshi et al., an increased prevalence of subacromial impingement syndrome was detected in individuals with increased kyphosis. This study demonstrated that increased kyphosis leads to decreased shoulder elevation and SD, resulting in impingement.^28^ that increased thoracic kyphosis causes the scapula to become more protracted and rotate downward. Excessive scapular protraction alters the role of the scapula in shoulder function, leading to potential compression under the acromion and subacromial tissues, including the subacromial bursa and rotator cuff.^16,28^ During AS, lumbar lordosis is decreased and thoracic kyphosis is increased, resulting in typical postural deterioration. We believe that in our patients, the fundamental cause of SD was postural changes that developed during the course of AS.

AS is a chronic inflammatory rheumatic disease characterized by axial involvement, with the sacroiliac joint being the initial affected area in all patients.^29^ Over time, the disease can progress upwards, sequentially affecting the lumbar, thoracic, and cervical spine. Progression of spinal involvement is also an indicator of disease severity. Inflammation and subsequent ankylosis lead to a decrease in spinal movements.^30^ Lumbar spine involvement may result in reduced measurements in the Schober test, whereas thoracic spine, costovertebral, and sternocostal joint involvement can restrict chest expansion. As kyphosis increases, the occiput-to-wall distance progressively increases.^31^ The current study demonstrated that in patients with AS and SD, radiological involvement in the lumbar and thoracic vertebrae was more prominent than in those without SD. In addition, chest expansion was significantly reduced in this group. Regression analysis revealed a significant correlation between the BASMI scores and SD. The BASMI score is an index used to assess the impact on spinal mobility in patients with AS, incorporating measurements such as lateral flexion, lumbar flexion, and tragus-to-wall distance in its subcomponents to detect reduced spinal mobility as a result of advanced disease.^32^ a high BASMI score indicates a more advanced stage of the disease. Therefore, a high BASMI score in participants with SD implies the presence of advanced disease, with expected lumbar and thoracic involvement and advanced sacroiliitis.^33^ During which the costovertebral joints participate in movement. In patients with AS, due to thoracic involvement, the chest expansion is usually limited.^34^ The observed limitation of chest expansion in the SD group may be explained by the higher thoracic involvement in this group. Moreover, as there was no significant difference in the total disease duration, the higher prevalence of thoracic involvement in the SD group indicated that patients in this group had a more severe disease. This was further supported by the higher prevalence of hip involvement, which is a poor prognostic indicator in the SD group. The sacroiliitis observed in the SD group was also more advanced, indicating the role of disease severity in the occurrence of SD. In Otoshi et al.’s study, an increase in the occiput-wall distance, an indirect indicator of kyphosis, was more pronounced in the impingement syndrome group.^28^ The tragus-wall distance, another indirect indicator of kyphosis, holds significant importance in the BASMI score. The high BASMI score in the SD group may be related to increased kyphosis, indicating advanced and severe disease.

We believe that our study will make a significant contribution to the literature, as it is the first study to investigate the presence of SD in patients with AS and to determine the factors associated with the occurrence of SD. Concerning the limitations of our study, cervical and shoulder pathologies in patients were ruled out through anamnesis, physical examination and ultrasonography. MRI could not be used to exclude cervical and shoulder pathologies because of the absence of complaints in the participants. Additionally, the lack of measurement of the radiological kyphosis angle in patients and the investigation of its relationship with SD can be considered another limitation.

## CONCLUSION

This study revealed the presence of SD in patients with AS and isolated axial involvement without shoulder or cervical pathology. Indicators of poor prognosis and advanced disease, such as sacroiliac radiographic findings and BASMI scores, were identified in the SD group. Therefore, investigating the presence of SD in patients with AS is important. If SD is present in these patients, the risk of developing shoulder pathologies increases, and early preventive rehabilitation measures should be implemented for appropriate treatment.
